# Hypocellularity in the Murine Model for Down Syndrome Ts65Dn Is Not Affected by Adult Neurogenesis

**DOI:** 10.3389/fnins.2016.00075

**Published:** 2016-03-02

**Authors:** Rosa López-Hidalgo, Raul Ballestín, Jessica Vega, José M. Blasco-Ibáñez, Carlos Crespo, Javier Gilabert-Juan, Juan Nácher, Emilio Varea

**Affiliations:** ^1^Neurobiology Unit and Program in Basic and Applied Neurosciences, Cell Biology Department, Universitat de ValènciaValència, Spain; ^2^Estructura de Recerca Interdisciplinar en Biotecnologia i Biomedicina (BIOTECMED), Universitat de ValènciaValència, Spain; ^3^Fundación Investigación Hospital Clínico de Valencia, INCLIVAValència, Spain; ^4^CIBERSAM, Spanish National Network for Research in Mental HealthValència, Spain; ^5^Genetics Department, CIBERSAM, Universitat de ValènciaValència, Spain

**Keywords:** adult neurogenesis, down syndrome, Ts65Dn mice, hippocampus, olfactory bulb, Ki67, doublecortin

## Abstract

Down syndrome (DS) is caused by the presence of an extra copy of the chromosome 21 and it is the most common aneuploidy producing intellectual disability. Neural mechanisms underlying this alteration may include defects in the formation of neuronal networks, information processing and brain plasticity. The murine model for DS, Ts65Dn, presents reduced adult neurogenesis. This reduction has been suggested to underlie the hypocellularity of the hippocampus as well as the deficit in olfactory learning in the Ts65Dn mice. Similar alterations have also been observed in individuals with DS. To determine whether the impairment in adult neurogenesis is, in fact, responsible for the hypocellularity in the hippocampus and physiology of the olfactory bulb, we have analyzed cell proliferation and neuronal maturation in the two major adult neurogenic niches in the Ts656Dn mice: the subgranular zone (SGZ) of the hippocampus and the subventricular zone (SVZ). Additionally, we carried out a study to determine the survival rate and phenotypic fate of newly generated cells in both regions, injecting 5′BrdU and sacrificing the mice 21 days later, and analyzing the number and phenotype of the remaining 5′BrdU-positive cells. We observed a reduction in the number of proliferating (Ki67 positive) cells and immature (doublecortin positive) neurons in the subgranular and SVZ of Ts65Dn mice, but we did not observe changes in the number of surviving cells or in their phenotype. These data correlated with a lower number of apoptotic cells (cleaved caspase 3 positive) in Ts65Dn. We conclude that although adult Ts65Dn mice have a lower number of proliferating cells, it is compensated by a lower level of cell death. This higher survival rate in Ts65Dn produces a final number of mature cells similar to controls. Therefore, the reduction of adult neurogenesis cannot be held responsible for the neuronal hypocellularity in the hippocampus or for the olfactory learning deficit of Ts65Dn mice.

## Introduction

Down Syndrome (DS) is the most common chromosomal aneuploidy, with an incidence of one in 1000 live births (Roizen and Patterson, 2003). This chromosomal alteration induces a variable phenotype that may include immune deficiencies, heart defects, increased risk of leukemia, and early development of Alzheimer's disease (Ball and Nuttall, 1980; Hof et al., 1995; Holland et al., 2000; Folin et al., 2003; Nadel, 2003; Lott and Head, 2005). The common feature among all DS subjects is the presence of intellectual disability reflected by impairment in learning and memory. Neural mechanisms underlying this alteration may include defects in the formation of neuronal networks, information processing and brain structural plasticity.

Brain plasticity can be defined as the ability to perform adaptive changes related to the structure and function of the central nervous system (Zilles, 1992). Structural plasticity takes place during both development and adulthood. During development, brain structural plasticity is a fundamental element that generates the specificity of connections present in the mature nervous system, allowing morphogenetic processes such as cell proliferation, cell migration, axonal or dendritic growth and remodeling. This plastic ability diminishes with age being limited during adulthood to some specific regions (Bonfanti, 2006). During adulthood, the neurogenic aspect of structural plasticity is limited to the subventricular zone (SVZ), and the subgranular zone (SGZ) of the dentate gyrus. Plastic processes are crucial for learning and adaptability (Cotman et al., 1998; Gage, 2000).

One of the cerebral regions where brain structural plasticity remains specially active during adulthood is the hippocampus (Leuner and Gould, 2010). In adult animals, the pyramidal neurons of the CA1 and CA3 regions, and the granule neurons of the dentate gyrus undergo dynamic modifications of their dendritic profiles and synaptic contacts. The generation of neurons persists in the dentate gyrus until old age (Altman, 1962; Altman and Das, 1965; Eriksson et al., 1998; Hastings and Gould, 1999; van Praag et al., 1999) and the formation of these neurons implies the growth of axons and dendrites and the generation of new synapses. During adulthood, neurons are generated from a population of stem cells that display astroglial characteristics (Seri et al., 2001; Garcia et al., 2004). In the hippocampus, after their generation, newly born cells migrate and differentiate into granule neurons. They will generate dendritic processes with spines (van Praag et al., 2002; Laplagne et al., 2006; Ribak and Shapiro, 2007; Toni et al., 2007), receiving synaptic inputs (Kaplan and Hinds, 1977; Markakis and Gage, 1999) and will extend their axons to specific targets (Hastings and Gould, 1999; Markakis and Gage, 1999; Toni et al., 2008), releasing glutamate as a main neurotransmitter (Toni et al., 2008). However, it is important to note that many of these newly born neurons in the hippocampus die during the process of maturation and integration (Gould et al., 1999; Dayer et al., 2003). Survival of newly born neurons during adulthood is highly sensitive to environmental stimulus and learning, suggesting that adult neurogenesis allows the individuals to adapt to new environments (Doetsch and Hen, 2005).

The other region displaying neurogenesis in the adulthood is the SVZ (for a review see Alvarez-Buylla and Lim, 2004), that gives rise to neurons that migrate through the rostral migratory stream (RMS) and integrate in the olfactory bulb. In the SVZ, slow cycling stem cells (also called B cells) express the astrocytic marker glial-fibrillary acidic protein (GFAP) and give rise to rapidly dividing intermediate progenitor cells (also called C cells) which stop expressing GFAP and begin expressing distal-less homeobox (Dlx)-2 (Alvarez-Buylla and Garcia-Verdugo, 2002). These progenitors produce neuroblasts (called type A cells) that express the polysialylated neural cell adhesion molecule (PSA-NCAM) and neuronal markers such as doublecortin (DCX) (Alvarez-Buylla and Garcia-Verdugo, 2002). Chains of neuroblasts leave the SVZ, moving along the RMS before terminating in the olfactory bulb where they differentiate into GABAergic interneurons (Alvarez-Buylla and Garcia-Verdugo, 2002; Doetsch, 2003; Mignone et al., 2004; Brazel et al., 2005; Merkle et al., 2007; Kriegstein and Alvarez-Buylla, 2009).

Several animal models that mimic the alterations in DS are available. One of the most studied is the Ts65Dn mouse. This model is segmentally trisomic for a portion of the mouse chromosome 16 that is orthologous to the long arm of the human chromosome 21. This segment contains approximately 140 genes, many of which are highly conserved between mice and humans (Gardiner et al., 2003; Sturgeon and Gardiner, 2011; Rueda et al., 2012). These mice display a delay in the acquisition of a number of sensory and motor tasks (Holtzman et al., 1996; Costa et al., 1999), as well as defects in learning and in the execution of memory tasks mediated by the hippocampus (Reeves et al., 1995; Holtzman et al., 1996; Demas et al., 1998, 1999; Escorihuela et al., 1998; Sago et al., 2000; Hyde et al., 2001), and deficits in long-term potentiation (LTP) (Siarey et al., 1997, 1999; Kleschevnikov et al., 2004). Many of these deficiencies may be consequence of impairments in neuronal structural brain plasticity and related to adult neurogenesis. In fact, abnormalities in the dendritic arborization of pyramidal neurons have been observed in the neocortex of DS individuals and mice models for this syndrome (Takashima et al., 1981, 1989; Kaufmann and Moser, 2000; Dierssen et al., 2003). Our group has demonstrated a similar atrophy of dendritic arborization in the granule neurons of the hippocampus although CA1 pyramidal neurons remained unaltered (Villarroya et al., unpublished results).

Ts65Dn mice display reduction in the rate of cell proliferation from early postnatal stages in the SGZ: by day 2 (Contestabile et al., 2007) or by day 6 (Lorenzi and Reeves, 2006). This impairment continues into adulthood, but its magnitude is a matter of controversy (Rueda et al., 2005; Clark et al., 2006; Contestabile et al., 2007). Several hypotheses have been proposed to explain the impairment in neurogenesis. In fact, the cell cycle progresses more slowly due to an arrest in G2 phase, and consequently it is produced a lower number of cells (Contestabile et al., 2007). DS individuals display a reduction in cell proliferation (Mittwoch, 1972) and a reduction in the number of differentiated neurons although the number of astrocytes remains unaltered (Guidi et al., 2008). *In vitro* studies have shown a reduction in neuronal production from neurospheres obtained from neuronal precursors extracted from human fetuses with DS (Bahn et al., 2002; Esposito et al., 2008). Finally, it has been observed an increment of apoptotic cells in the hippocampus of DS fetuses (Guidi et al., 2008). Altogether, reduced cell proliferation and increased apoptosis may generate the hypocellularity observed in the brain of DS individuals (Guidi et al., 2008), or the lower number of dentate gyrus granule cells in Ts65Dn mice (Insausti et al., 1998; Lorenzi and Reeves, 2006). Studies in the SVZ, the RMS and the olfactory bulb have revealed a reduction in cell proliferation in the Ts65Dn mice model (Chakrabarti et al., 2011; Bianchi et al., 2014). Moreover olfactory learning is impaired in Ts65Dn mice (de Souza et al., 2011), similarly to the observed impairment in olfactory performance in adult with DS (Nijjar and Murphy, 2002). Activation of the olfactory system by odor exposition doesn't affect the number of proliferating cells; however the number of survival cells in the olfactory bulb is increased (Rochefort et al., 2002). This effect is different to the one observed in the hippocampus where the learning process, as it happens in an enriched environment, increases cell proliferation (reviewed in Kempermann et al., 2004).

In this study, we aim to characterize the processes of cell proliferation and neuronal maturation in the two main neurogenic regions of adult Ts65Dn mice: the SGZ and the SVZ (and also the RMS). We also want to characterize the survival rate and phenotype of the surviving cells in the hippocampus and the olfactory bulb of the adult Ts65Dn mice, in order to analyze whether these processes could be responsible for the hypocellularity and hypofunction observed in these two regions of this DS model.

## Materials and methods

Experimental mice were generated by repeated backcrossing of Ts65Dn females to C57/6Ei 9 C3H/HeSnJ (B6EiC3) F1 hybrid males. The parental generation was obtained from the research colony of Jackson Laboratory. Euploid littermates of Ts65Dn mice served as controls. We have used a total of 17 trisomic mice and 24 euploid mice. For the characterization of proliferation and maturation we have used 4- to 5-month-old male mice (10 trisomic mice and 16 euploid mice). For the study of survival and characterization of newly born cells we have used 4-month old male mice (7 trisomic mice and 8 euploid mice). Mice were injected with 5′BrdU (50 mg/kg i.p.) twice per day (one injection every 12 h) during 2 days and were sacrificed 21 days after the last injection.

The genotypic characterization was established by qRT-PCR using SYBR Green PCR master mix (Applied Biosystems) from genomic DNA extracted from mice tails by mean of the phenol-chloroform method. The relative amount of each gene was quantified by the ABI PRISM 7700 (Applied Biosystems). The genes analyzed where APP (3 copies) and Apo-B (2 copies) as previously described (Liu et al., 2003; Hernández et al., 2012). All animal experimentation was conducted in accordance with the Directive 2010/63/EU of the European Parliament and of the Council of 22 September 2010 on the protection of animals used for scientific purposes and was approved by the Committee on Bioethics of the Universitat de València. Every effort was made to minimize the number of animals used and their suffering.

Animals were transcardially perfused under deep anesthesia (choral hydrate 4%, 1 ml/100 gw) using a solution containing 4% paraformaldehyde in phosphate buffer 0.1 M, pH 7.4. Brains were removed and cryoprotected using 30% sucrose. Fifty microns thick sections (6 subseries were collected for each brain) were obtained using a sliding freezing microtome.

### Immunohistochemical procedure

Tissue was processed “free-floating” for immunohistochemistry as follows. Briefly, sections were incubated with 10% methanol and 3% H2O2 in phosphate-buffered saline (PBS) for 10 min to block endogenous peroxidase activity.

After this, sections were treated for 1 h with 5% normal donkey serum (NDS) (Jackson ImmunoResearch Laboratories, West Grove, PA, USA) in PBS with 0.2% Triton- X100 (Sigma-Aldrich, St Louis, MO, USA) and were incubated overnight at room temperature either in polyclonal rabbit IgG anti-Ki67 (1:1000, Novocastra, NCL-Ki67p), polyclonal goat IgG anti-DCX (1:200, Santa Cruz, sc-8066), monoclonal mouse IgM anti-PSA-NCAM (1:700, Chemicon, MAB5324) or polyclonal goat IgG anti-NeuroD (1:1000; Santa Cruz, sc-1084) antibodies. After washing, sections were incubated for 2 h with donkey anti-rabbit IgG, donkey anti-mouse IgM or donkey anti-goat IgG biotinylated antibodies (1:250; Jackson ImmunoResearch Laboratories, West Grove, PA, USA), followed by avidin–biotin–peroxidase complex (ABC; Vector Laboratories, Peterborough, UK) diluted in PBS, for 30 min. Color development was achieved by incubating with 0.05% 3,3-diaminobenzidine tetrahydrochloride (Sigma-Aldrich) and 0.033% hydrogen peroxide in PB for 4 min. Finally, sections were mounted on slides, dried for 1 day at room temperature, dehydrated with ascending alcohols and rinsed in xylene. After this, sections were coverslipped using Eukitt mounting medium (PANREAC). All studied sections passed through all procedures simultaneously in order to minimize any difference from the immunohistochemical staining itself. To avoid any bias in the analysis, all slides were coded prior to analysis and remained so until the experiment was completed.

### Detection of apoptotic cells (cleaved caspase-3 positive)

Tissue was processed “free-floating” for immunofluorescence as follows. Briefly, sections were incubated with citrate buffer (0.01M, pH 6.0) for 1 min at 100°C. After this, sections were treated for 1 h with 5% normal donkey serum (NDS) (Jackson ImmunoResearch Laboratories, West Grove, PA, USA) in PBS with 0.2% Triton- X100 (Sigma-Aldrich, St Louis, MO, USA) and were incubated overnight at room temperature in polyclonal rabbit IgG anti-cleaved caspase-3 (1:500, Cell Signaling, 9661) antibody. After washing, sections were incubated for 2 h with donkey anti-rabbit IgG conjugated with DL549 (1:200, Jackson Immunoresearch) for 2 h. After washing, sections were mounted on slides using Dako fluorescent medium (Dako North America, California). The sections were counterstained with 4′, 6-Diamidino-2-phenylindole (DAPI) (dilution 1/500) (Sigma-Aldrich, St Louis, MO, USA) to identify cellular nuclei. The analysis of sections was performed using a confocal microscope (Leica TSC-SPE) using 40X objective. Serial stacks of the different regions (granule layer of dentate gyrus, RMS and olfactory bulb) were analyzed using ImageJ software. Interval between z planes was 1.15 μm.

### Cell quantification of proliferating, immature neurons, and apoptotic cells

We have analyzed the number of cells proliferating (Ki67 positive), the number of neurons in process of maturation (DCX positive) and the number of caspase 3 positive cells in two regions: the SGZ of the dentate gyrus in the hippocampus and the SVZ and RMS. Moreover we have analyzed the number of neurons in process of maturation using other markers, NeuroD and PSA-NCAM in the SGZ.

For the SGZ of dentate gyrus we have estimated the total number of cells expressing these markers. The number of cells expressing these markers in each region was estimated using a modified version of the fractionator method (West, 1993) as described before (Nacher et al., 2002). We counted cells covering 100% of the sample area, that is, within each section, all labeled cells were counted. The fractionator sampling scheme refers to the methodology of examining one out of every 6 brain sections. Thus, our modification of the optical dissector combined with a 1:6 fractionator sampling is truly a modification of the optical fractionator method. 1:6 systematic series of sections covering the whole rostral to caudal extension of this structure were viewed under an Olympus CX41 microscope. Cell somata were identified and counted with a 40X objective (NA 0.65). Cells appearing in the upper focal plane were omitted to prevent overcounting. The volume of the different areas analyzed was determined for each animal using the Cavalieri's principle (Gundersen and Jensen, 1987).

For the SVZ, RMS and olfactory bulb we have analyzed the cell density. We have analyzed 3–4 sections per animal covering the whole rostral to caudal extension (Bregma 1.3, 0.98, 0.5, and 0.14 mm, for SVZ and RMS and Bregma 4.28, 3.92, and 3.56 mm for the olfactory bulb). Cells were observed under an Olympus CX41 microscope. Cell somata were identified and counted with a 40X objective. The volume of the different areas analyzed, as pointed out before, was determined for each animal using the Cavalieris's principle (Gundersen and Jensen, 1987). The areas of the analyzed regions were measured using ImageJ software (NIH). Results were expressed as cells per square millimeter. Ratio values (DCX/Ki67 and RMS/SVZ) were obtained for each animal. For the case of caspase 3 positive cells, due to the low number of cells presents, we have counted all the cells presents in the regions analyzed in one to six sections. Data were reported as mean ± sem. Means were determined for each experimental group and the data were statistically analyzed using the SPSS software package (version 15). Differences between groups were analyzed with one way ANOVA followed by Student–Newman–Keuls *post-hoc* test. Nissl-stained sections adjacent to the measured ones were used to determine the location and borders of the analyzed regions.

### Phenotipic characterization of newly born cells

In order to characterize the phenotype of the newly born cells in the dentate gyrus and the olfactory bulb, we have performed a triple immunolabeling using antibodies against 5′BrdU, NeuN (neurons) and GFAP (astrocytes). The triple labeling was performed following the “free-floating” procedure described previously with some changes.

Sections were incubated in PBS during 60 min to 60°C. After cooling down, sections were incubated with HCl 2N for 30 min. After washing, sections were incubated with a blocking solution containing 10% NDS for 1 h and overnight with a mix of monoclonal rat IgG anti-5′BrdU (1:1000, Inmunological Direct, OBT0030), polyclonal rabbit IgG anti-GFAP (1:500 Sigma Aldrich, G9269) and monoclonal mouse IgG anti-NeuN (1:100, Millipore, MAB377) antibodies. After washing, sections were incubated with a mix of secondary antibodies containing: Donkey anti-rat IgG conjugated with Alexa 488 (1:200, Molecular Probes), donkey anti-rabbit IgG conjugated with DL649 (1:200, Jackson Immunoresearch) and donkey anti-mouse IgG conjugated with DL549 (1:200, Jackson Immunoresearch) for 2 h. After washing, sections were mounted on slides using Dako fluorescent medium (Dako North America, California). The analysis of sections was performed using a confocal microscope (Leica TSC-SPE) using 40X objective. Stacks (z-step 1.15 μm) of the different regions (granule layer of dentate gyrus and olfactory bulb) and were analyzed using ImageJ software. A minimum of 50 cells were analyzed for each animal. Percentages for every marker were obtained and mean for each group and region was determined and analyzed using SPSS software.

## Results

We analyzed the cell proliferation, neuronal maturation and phenotype of the surviving cells in the two major neurogenic areas during adulthood, the SGZ of the dentate gyrus (including the granule layer for the analysis of cell survival) and the SVZ (including here also the RMS and the olfactory bulb for the analysis of cell survival).

### Subgranular zone and dentate gyrus

**Figure 1:**
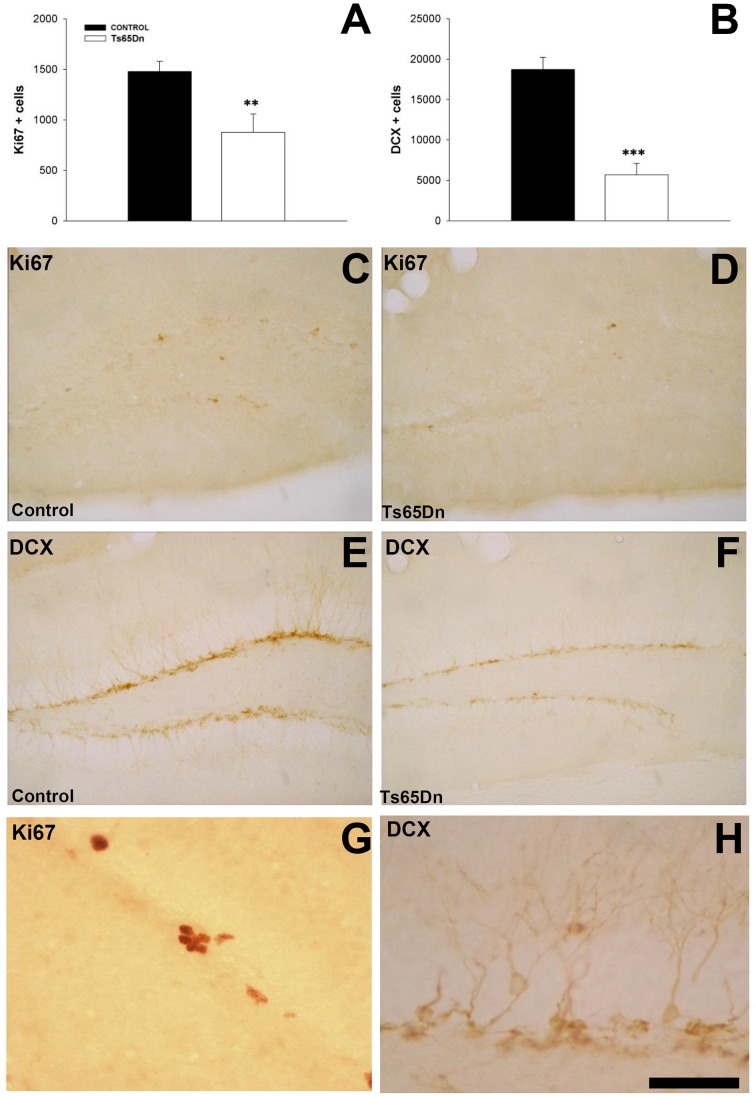
**Proliferating cells and immature neurons in the dentate gyrus of adult Ts65Dn mice. (A)** Graph showing the changes in the number of proliferating cells (Ki67) between control (black bar) and Ts65Dn mice (white bar). **(B)** Graph showing the change in the number of immature neurons (DCX) in the dentate gyrus between control (black bar) and Ts65Dn mice (white bar). Representative images of the expression of Ki67 in the SGZ of control **(C)** and Ts65Dn mice **(D)** and DCX in the dentate gyrus of euploid **(E)** and Ts65Dn mice **(F)** Details of the expression of Ki67 **(G)** and DCX **(H)** in control mice. Scale bar: 250 microns **(B–F)** and 50 microns **(G,H)**. ^**^p < 0.01; ^***^p < 0.001).

We studied the number of proliferating cells using the marker Ki67 (Figures 1A,C,D). In the SGZ we observed that Ki67 labeled nuclei often appeared in clusters (Figures 1C,D). Trisomic mice displayed a reduction in the number of proliferating cells in the SGZ (878 ± 179 cells in the SGZ of Ts65Dn mice vs. 1479 ± 101 cells in the euploid littermates, *p* < 0.01) (Figure 1A).

**Figure 2:**
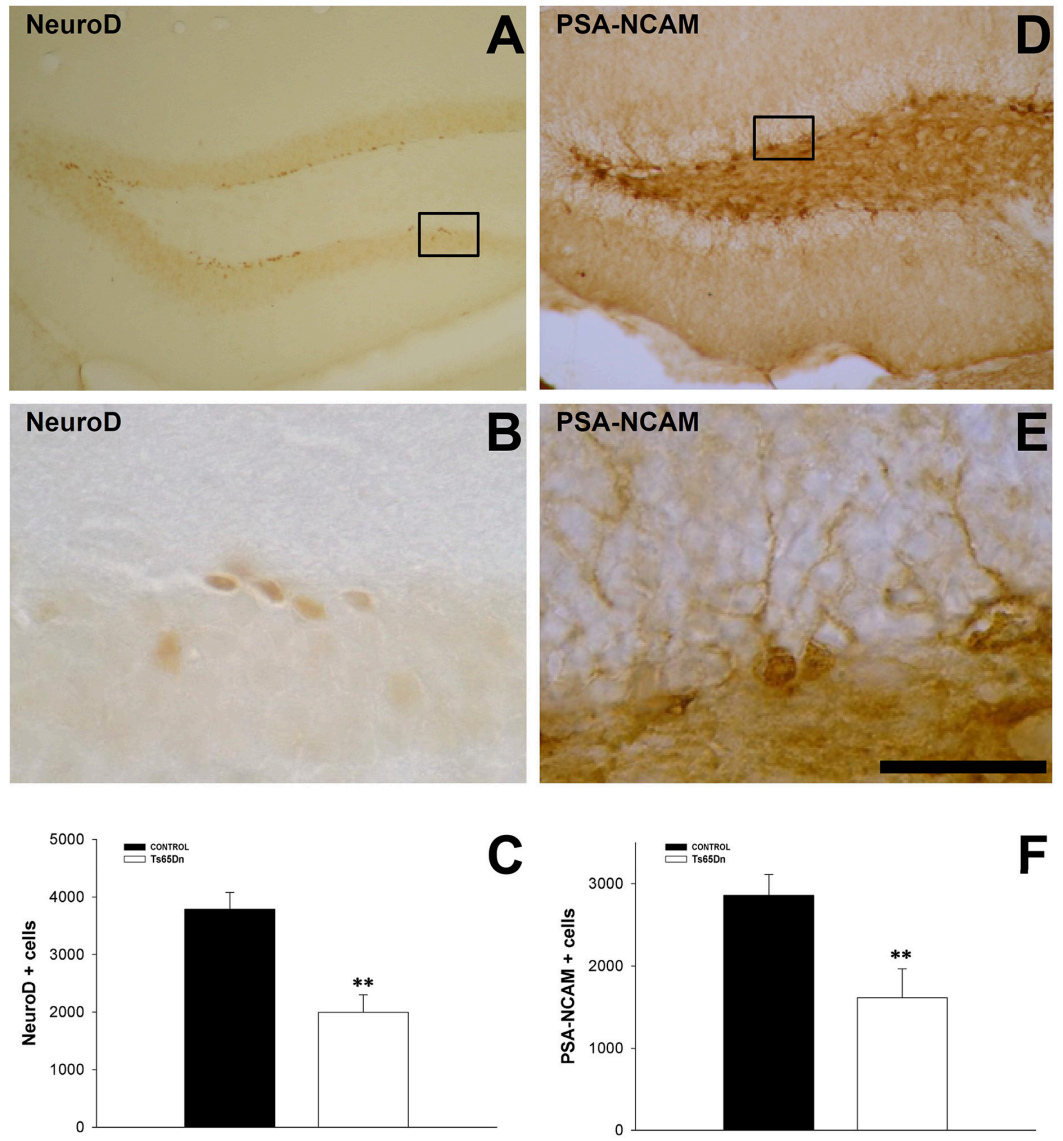
**Immature neurons in the dentate gyrus of adult Ts65Dn mice. (A)** Representative image of the expression of NeuroD in the SGZ. **(B)** High magnification image of the region boxed in **(A)**. **(C)** Graph showing the changes in the number of immature neurons in the SGZ (NeuroD) between control (black bar) and Ts65Dn mice (white bar). **(D)** Representative image of the expression of PSA-NCAM in the SGZ. **(E)** High magnification image of the region boxed in **(D)**. **(F)** Graph showing the changes in the number of immature neurons in the SGZ (PSA-NCAM) between control (black bar) and Ts65Dn mice (white bar). Scale bar: 350 microns for **(A,D)** and 75 microns for **(B,E)**. ^**^p < 0.01.

We quantified the number of immature neurons using the marker doublecortin (DCX) (Figures 1B,E,F). This marker is present in the cytoplasm, both in the cell body and the proximal dendrites of newly generated neurons. DCX positive cells are located in the inner part of granule layer adjacent to the SGZ, where neurons are produced (Figures 1E,F). We have observed a reduction in the number of immature neurons in the dentate gyrus of the Ts65Dn model (5687 ± 1408 in Ts65Dn mice vs. 18713 ± 1518 in their euploid littermates, *p* < 0.001) (Figure 1B). Similar results were obtained with other markers for immature neurons such as the transcription factor NeuroD (Figures 2A–C), a protein involved in the differentiation of hippocampal granule neurons (Schwab et al., 2000), which is also expressed in some progenitor cells in the rodent SGZ (Seri et al., 2004), or the polysialylated form of the Neural Cell Adhesion Molecule (PSA-NCAM (Figures 2D–F). For NeuroD, we have observed a moderate but significant reduction in the number of immature neurons (1998 ± 303 in the Ts65Dn mice vs. 3788 ± 291 in their euploid littermates, *p* < 0.01) (Figure 2C) or the polysialylated form of the Neural Cell Adhesion Molecule (PSA-NCAM) (1616 ± 350 immature neurons in the granule layer of Ts65Dn mice vs. 2857 ± 254 immature neurons in their euploid littermates, *p* < 0.01) (Figure 2F).

**Figure 3:**
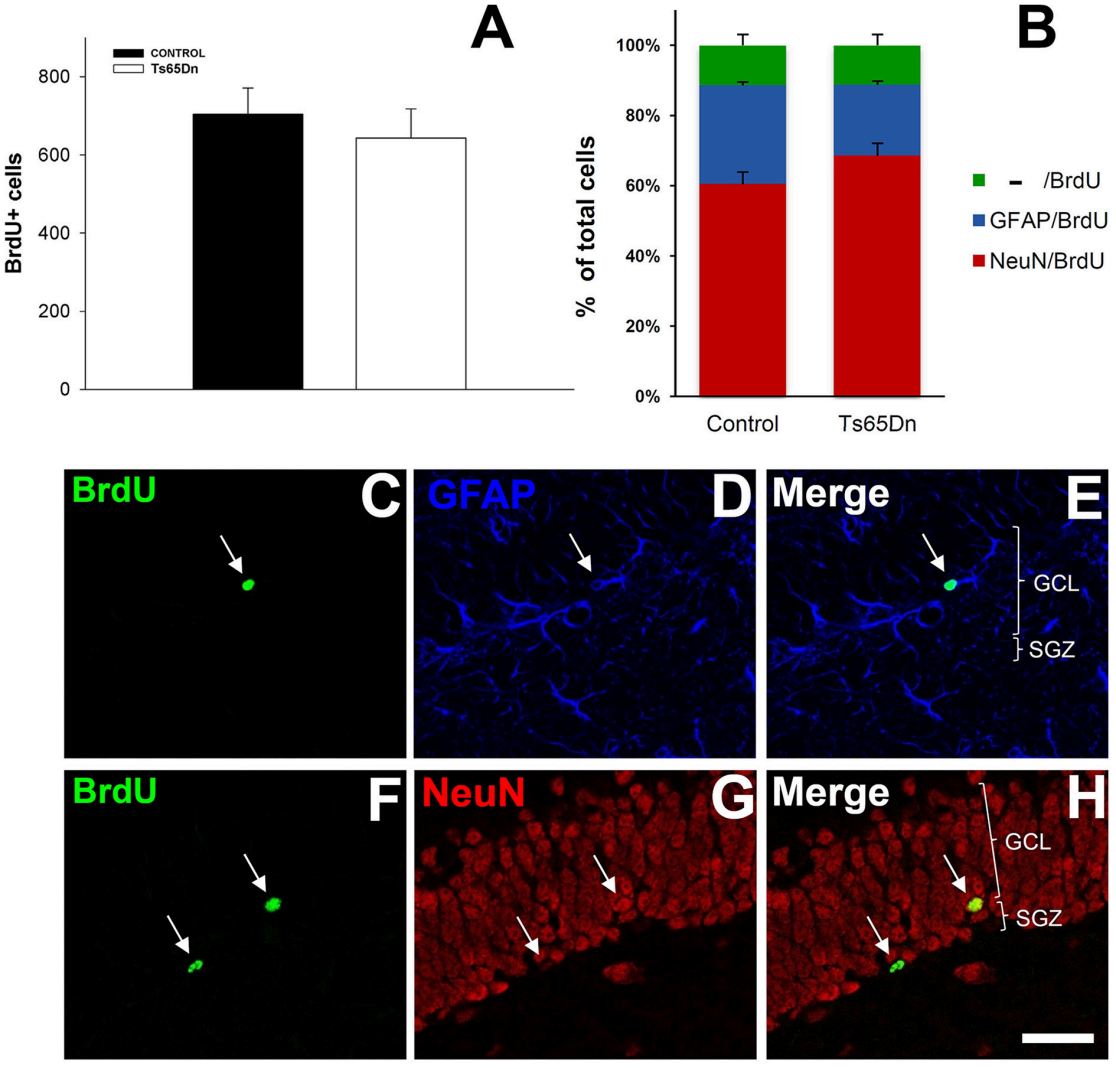
**Number and phenotype of surviving cells in the granule layer of the dentate gyrus of the adult Ts65Dn mice. (A)**. Graph showing the total number of 5′BrdU positive cells in the granule layers of control (black bar) and Ts65Dn mice (white bar). **(B)** Graph showing the phenotype of the surviving cells in the granule layer of dentate gyrus: NeuN/BrdU positive cells (red bar), GFAP/BrdU positive cells (blue bar) and BrdU-only positive cells (green bar). **(C–H)** Representative confocal images showing colocalization between BrdU and GFAP **(C–E)** and BrdU and NeuN **(F–H)**. Scale bar: 50 microns.

We analyzed the number of surviving cells from this population of newly generated cells using 5′BrdU injection and allowing mice to survive for 21 days (Figure 3). After this period, we analyzed the number of cells that had incorporated 5′BrdU. We observed that, in the hippocampus, labeled nuclei were mainly restricted to the granule layer. The quantification of the total number of surviving cells in the granule layer of the dentate gyrus reflected no changes between euploid and Ts65Dn mice (Figure 3A). Therefore, despite the difference in cell proliferation, the percentage of cells surviving is higher in Ts65Dn mice. We observed 643 ± 75 cells in the Ts65Dn granule layer and 704 ± 67 in the granule layer of the euploid littermates (*p* = 0.57). Using triple immunohistochemistry, we have characterized the phenotype of the surviving cells (Figures 3B–H). Surviving cells were characterized mainly as neurons (NeuN+) (60.4% in euploid mice and 68.6% in Ts65Dn mice), whereas those idenfied as astrocytes (GFAP+) represented less than one third of them (28.2% in euploid mice and 20.2% in Ts65Dn mice). The rest of cells were negative for GFAP or NeuN and their percentages were similar in both groups (11.4% in euploid mice and 11.2% in Ts65Dn mice). There were no statistical differences between groups.

### Subventricular zone, rostral migratory stream, and olfactory bulb

**Figure 4:**
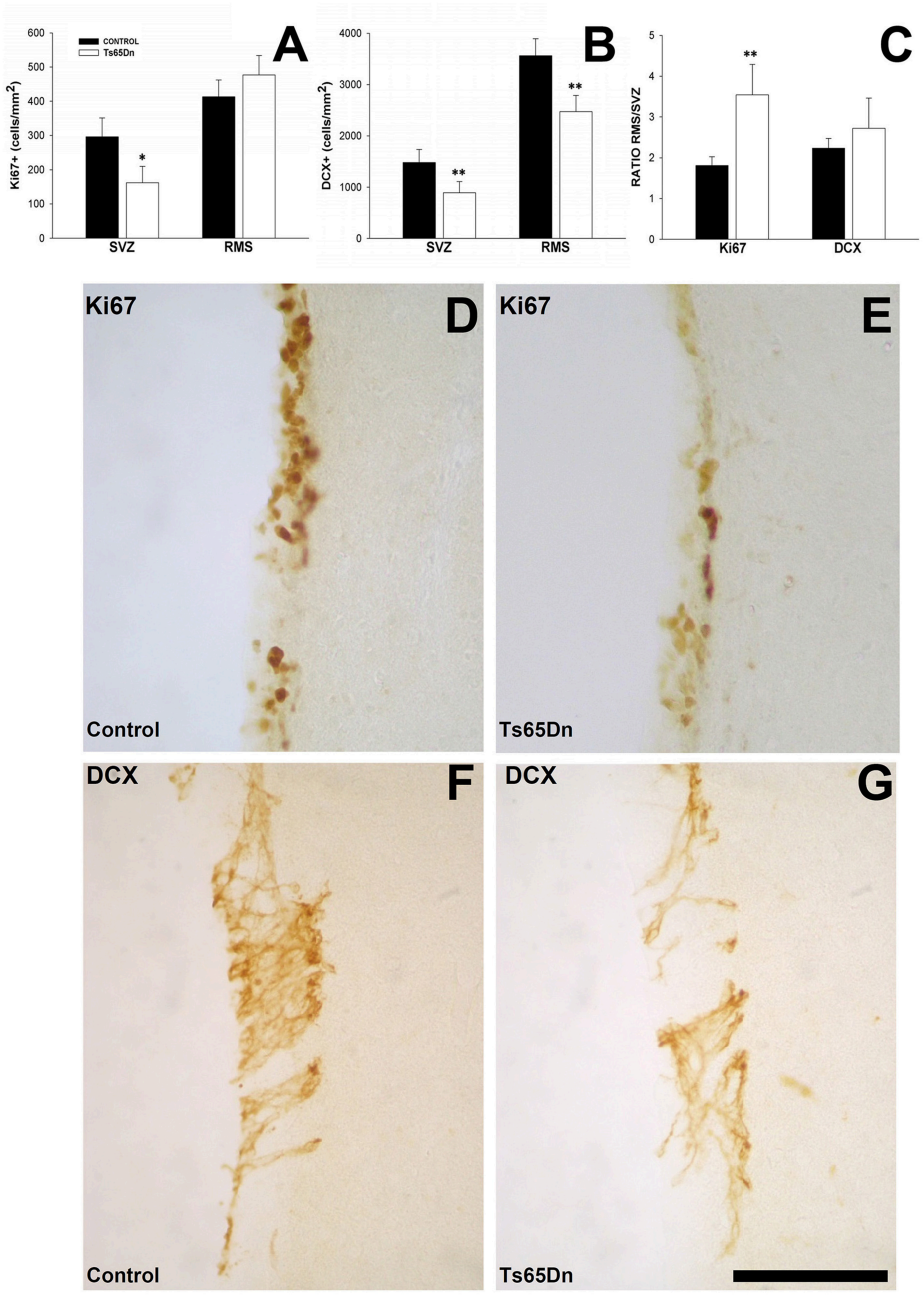
**Proliferating cells and immature neurons in the SVZ and the RMS of adult Ts65Dn mice. (A)** Graph showing the changes in the density of proliferating cells (Ki67) in the SVZ and RMS between control (black bar) and Ts65Dn mice (white bar). **(B)** Graph showing the change in the density of immature neurons (DCX) in the SVZ abd RMS between control (black bar) and Ts65Dn mice (white bar). **(C)** Graph showing the ratio of proliferative and immature neurons between the SVZ and RMS. Representative images of the expression of Ki67 in the SVZ of control **(D)**, and Ts65Dn mice **(E)** and DCX in the SVZ of euploid **(F)** and Ts65Dn mice **(G)**. Scale bar: 100 microns. ^*^p < 0.05; ^**^p < 0.01).

We studied the density of cells proliferating in the SVZ and proximal region of RMS using the marker Ki67. The volume analysis revealed no changes between control and trisomic mice in the SVZ (0.34 ± 0.01 mm^3^ for euploid mice vs. 0.32 ± 0.02 mm^3^ for trisomic mice) neither in the RMS (0.61 ± 0.03 mm^3^ in euploid mice vs. 0.58 ± 0.06 mm^3^ for Ts65Dn mice). We observed clusters of stained nuclei in the SVZ and in the RMS (Figures 4D,E). Trisomic mice displayed a reduction in the density of proliferating cells in the SVZ (Figure 4A) (162 ± 48 cells/mm^2^ in Ts65Dn mice vs. 296 ± 55 cells/mm^2^ in their euploid littermates, *p* < 0.05). However, when we quantified the density of proliferating cells in the proximal RMS (Figure 4A), we failed to observe differences between groups (413 ± 49 cells/mm^2^ in Ts65Dn mice vs. 477 ± 57 cells/mm^2^ in their euploid littermates, *p* = 0.4714). We also analyzed the ratio between the density of proliferating cells in the SVZ and the RMS (Figure 4C). We observed that trisomic mice displayed a higher proportion of proliferating cells in the RMS vs. the SVZ than their euploid littermates (3.54 ± 0.75 times for Ts65Dn mice vs. 1.81 ± 0.22 times for their euploid littermates, *p* < 0.01). This result suggests that cells proliferate slowly in the trisomic mice and that the proliferative process continues into the RMS during the migrating process in a higher proportion than in the control mice.

We quantified the density of immature neurons in the SVZ and the RMS using the marker DCX. This molecule is present in the cytoplasm in both the cell body and the proximal dendrites of newly generated neurons (Figures 4F,G). We observed a reduction (Figure 4B) in the number of immature neurons in the SVZ of the Ts65Dn model (891 ± 221 cells/mm^2^ in the SVZ of the Ts65Dn mice vs. 1483 ± 253 cells/mm^2^ in their euploid littermates, *p* < 0.01). When we analyzed the density of immature neurons in the RMS of the Ts65Dn mice (Figure 4B), we have also observed a reduction in their density (2472 ± 315 cells/mm^2^ in the RMS of the Ts65Dn mice vs. 3564 ± 332 cells/mm^2^ in the RMS of their euploid littermates, *p* < 0.01). We checked also the ratio between immature neurons in the SVZ and the RMS (Figure 4C), in this case there were not differences between groups (2.72 ± 0.74 for the Ts65Dn mice vs. 2.23 ± 0.24 for their euploid littermates).

The analysis of the ratio between proliferating (ki67) and immature (DCX) cells in SVZ reflected no differences (5.02 ± 0.34 DCX cells for each Ki67 cell in controls vs. 5.55 ± 1.23 in Ts65Dn mice), however when we analyzed the same ratio in the RMS there were higher differences (7.69 ± 0.95 DCX cells for each Ki67 cell in controls vs. 6.25 ± 1.42 in Ts65Dn mice) but still not statistically significant.

**Figure 5:**
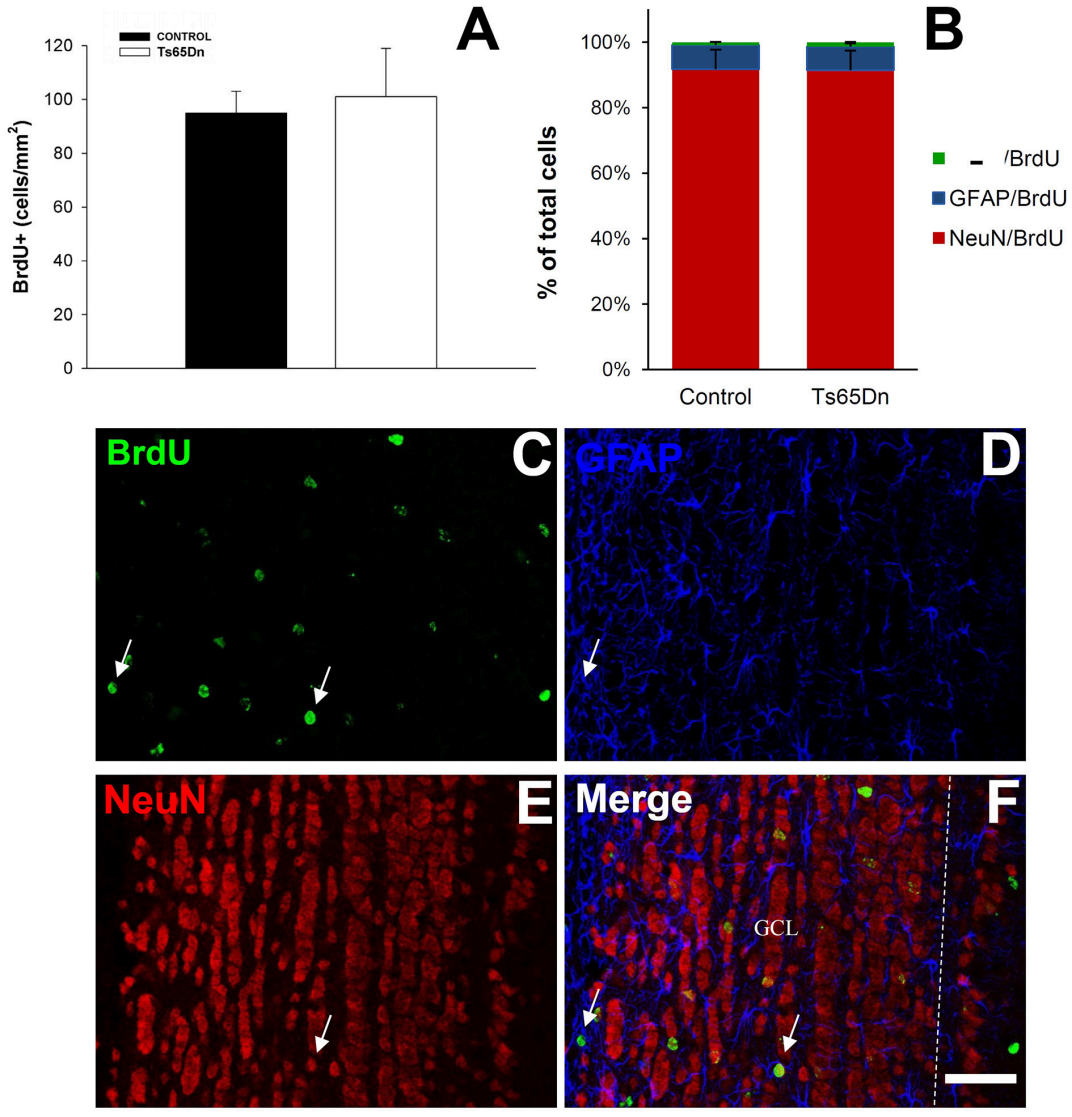
**Number and phenotype of surviving cells in the olfactory bulb of the adult Ts65Dn mice. (A)** Graph showing the density of BrdU positive cells in the olfactory bulb of control (black bar) and Ts65Dn mice (white bar). **(B**) Graph showing the phenotype of surviving cells in the olfactory bulb: NeuN/BrdU positive cells (red bar), GFAP/BrdU positive cells (blue bar) and BrdU only positive cells (green bar). **(C–F)** Representative confocal images showing colocalization between BrdU **(C)**, NeuN **(D)** and GFAP **(E)** and the merged image **(F)** arrow in **(D)** show a neuron and in E an astrocyte. Dashed line marks the limit of granule layer. Scale bar: 50 microns.

As described before, we analyzed the survival of the adult generated cells using 5′BrdU injection and allowing mice to survive for 21 days. After this period we analyzed the density of 5′BrdU cells (Figure 5A). We observed that, in the olfactory bulb they were present in the granule and glomerular layers. The quantification of the density of surviving cells in the olfactory bulb reflected no changes between euploid and Ts65Dn mice (Figure 5A). We have observed 101 ± 18 cells/mm^2^ in the olfactory bulb of the Ts65Dn mice and 95 ± 8 cells/mm^2^ in the olfactory bulb of their euploid littermates (*p* = 0.73). The phenotypical characterization of the surviving cells using triple immunofluorescence (Figures 5C–F) revealed that they were mainly neurons (91.8% in euploid mice and 91.5% in Ts65Dn mice) (Figure 5B), whereas astrocytes represented less than one tenth of surviving cells (7.4% in euploid mice and 7.2% in Ts65Dn mice). The rest of the cells were negative for GFAP or NeuN and their percentage was similar in both groups (0.8% in euploid mice and 1.3% in Ts65Dn mice). As it happened in the hippocampus, there were no statistical differences between groups.

### Apoptotic cells in the hippocampus, RMS and olfactory bulb

**Figure 6:**
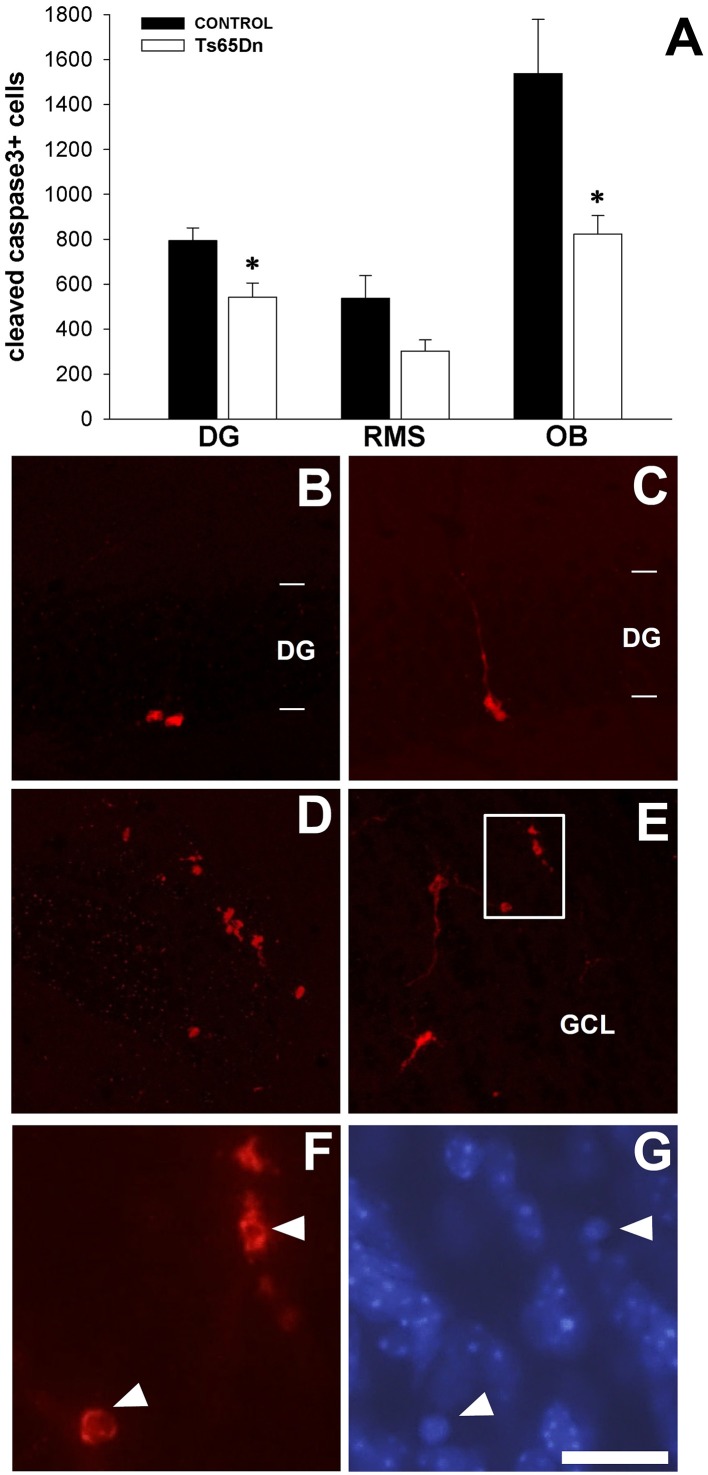
**Apoptotic cells (cleaved-caspase 3 positive cells) in the dentate gyrus of the hippocampus and the olfactory bulb of the adult Ts65Dn mice. (A)** Graph showing the changes in the number of cleaved-caspase 3 cells in the dentate gyrus (DG), RMS and olfactory bulb (OB) between control (black bar) and Ts65Dn mice (white bar). Representative images of the expression of cleaved-caspase 3 positive cells in the dentate gyrus **(B,C)**. **(D)** Cleaved-caspase 3 positive cells along the RMS, observe their morphological aspect. **(E)** Apoptotic cells in the granule layer of the olfactory bulb (GCL). **(F)** High magnification image of the region boxed in **(E)**. **(G)** DAPI counterstaining of the area showed in **(F)**. Observe the presence of condensed nuclei in the cells displaying cleaved caspase 3 expression (arrowheads). Images are taken from a Ts65Dn animal, presenting similar morphologies in controls. Scale bar: 75 microns **(B–E)** and 20 microns **(F–G)**. ^*^p < 0.05.

Cell proliferation is impaired in SGZ and SVZ in the Ts65Dn mice model, however there are no differences in the final number of surviving cells (BrdU+). To understand this paradox, we analyzed the number of apoptotic cells (positive for cleaved caspase 3) in the dentate gyrus of the hippocampus, the RMS and the olfactory bulb (Figure 6). In the dentate gyrus, we have observed cells with different morphologies (Figures 6B,C). Cleaved caspase 3 cells have been also observed in the RMS (Figure 6D) and in the granular layer of the olfactory bulb (Figure 6E). We quantified the number of cells positive for cleaved caspase 3 in the different regions (Figure 6A). There was a lower number of apoptotic cells in the dentate gyrus of the Ts65Dn mice model (542 ± 63 cells in the dentate gyrus of Ts65Dn mice vs. 794 ± 56 in their euploid litteramates, *p* < 0.05). In the olfactory system, we quantified the number of apoptotic cells in the RMS and the olfactory bulb. For the RMS we observed a lower number but not statistically significant (302 ± 50 cells in the RMS of the Ts65Dn mice vs. 537 ± 101 for their euploid littermates, *p* = 0.067). In the olfactory bulb, we observed a lower number of apoptotic cells in the Ts65Dn mice model (823 ± 82 cells in the olfactory bulb of the Ts65Dn mice model vs. 1538 ± 241 for their euploid littermates, *p* < 0.05).

## Discussion

In this study, we analyzed the proliferation and maturation rate in the SGZ, SVZ, and RMS of the DS mice model Ts65Dn and the integration of the newly born cells in the dentate gyrus and olfactory bulb. We have observed a reduction in the number of (Ki67 positive) proliferative cells in the SGZ and SVZ. The analysis of the number of (DCX positive) immature neurons indicated a decrease in the Ts65Dn mice in all regions analyzed (SGZ, SVZ, and RMS). The analysis of the number of apoptotic cells, reflects that Ts65Dn mice have a lower level of cells death in all the regions analyzed. Moreover, we analyzed the number of surviving cells and their phenotype 21 days after their generation using intraperitoneal injections of 5′BrdU. The absolute number of surviving cells was similar in both groups; their characterization reflected that the majority of them were neurons in both trisomic and euploid mice, and the proportion of them did not change between groups. So, we can conclude that Ts65Dn mice display impairment in cell proliferation during adulthood, but due to the reduced apoptotic rate, the number of cells surviving after 21 days is similar in both groups and their phenotype is similar in Ts65Dn mice and their euploid littermates.

First of all, we analyzed the density (SVZ and RMS) and the total number of proliferating cells (DG) in the adulthood using the marker Ki67; this protein is present during G1, S, G2 and mitosis phases but it is absent during G0 phase (Gerdes et al., 1984). We have observed a 41% reduction in cell proliferation in the adult SGZ. Our results are in accordance with previous studies showing a reduction in cell proliferation in this region (Clark et al., 2006). However, other studies have found that the number of proliferating cells is maintained in the SGZ of adult Ts65Dn mice (Rueda et al., 2005). This discrepancy could be explained because of the fine regulation of adult neurogenesis and the high sensitivity to external and internal factors that could induce drastic changes in cell proliferation (for a detailed review see Balu and Lucki, 2009). In Rueda's study (Rueda et al., 2005), mice are subjected to BrdU injection during 12 days and surviving neurons are quantified at 30 days. They observe changes in old but no young animals. Our study has been performed in mouse of a similar age but our procedure is different (2 days BrdU injection, 21 days survival). We have observed an impairment in cell proliferation and this study observe no change. Perhaps the manipulation during 12 days has induced stress of mice and trisomic mice are less sensitive to this manipulation. Supporting this possibility our group has observed a lower sensitivity (analyzed as a reduction in cell proliferation) to manipulation in trisomic mice than control (Ballestín et al., unpublished results).

Rodents living in an enriched environment show increased the number of surviving cells in the SGZ (van Praag et al., 1999). However, stress conditions reduce drastically neurogenesis in the SGZ (for a review see Mirescu and Gould, 2006). Human DS fetuses also displayed a reduction in the cell proliferation in the SGZ (Contestabile et al., 2007).

The analysis of cell proliferation in the SVZ and RMS of the Ts65Dn mice reflects a reduction in the SVZ (46%) but no change in the RMS. Previous studies have shown similar results in 15 days-old mice (Bianchi et al., 2014). However, when the animals analyzed were old (13 months old) both regions displayed a similar reduction in cell proliferation (Bianchi et al., 2014). The animals used in our study are adults (4–5 months old) and consequently our results suggest that, at this age, the alterations in cell proliferation are similar to those observed in younger animals. Moreover, we have analyzed the ratio of this parameter in the RMS/SVZ in Ts65Dn and control mice and we have observed that it is higher in the trisomic mice. Our results support previous studies that claim that the proliferation process is slower in Ts65Dn mice (Contestabile et al., 2007). When we compared the ratio between proliferating (Ki67) and maturating (DCX) in SVZ we observed that there were no differences (5.02 ± 0.34 DCX cells for each Ki67 cell in controls vs. 5.55 ± 1.23 in Ts65Dn mice), however when we analyzed the same ratio in the RMS there is a trend toward a higher ratio in the control mice (7.69 ± 0.95 DCX cells for each Ki67 cell in controls vs. 6.25 ± 1.42 in Ts65Dn mice). It has been previously characterized that the cell cycle last longer (at least its G2 phase) in trisomic mice (Contestabile et al., 2007). Therefore, more cells could reach the RMS while they are still proliferating. The reason for this enlarged cell cycle can be connected with the fact that microtubules are altered in this model and this alteration may compromise the formation of the mitotic spindle (Liu et al., 2008).

In the second part of our study, we have analyzed the alteration in the number of immature neurons in the SGZ, SVZ, and RMS. We have used the marker doublecortin (DCX). This protein acts as a microtubule associated protein (Moores et al., 2003). DCX is transiently expressed by immature neurons and is absent from glial cells (Rao and Shetty, 2004); it participates in neuronal migration (Feng and Walsh, 2001; Brown et al., 2003; Couillard-Despres et al., 2005). The analysis of the DCX cells in the different regions showed a reduction in number and density of positive cells in the Ts65Dn mice in all the regions analyzed (70% in the SGZ, 40% in the SVZ and 31% in the RMS).

There is a discrepancy between the reduction in cell proliferation (41%) and that of immature neurons (70%) in the SGZ. There are, at least, four possible explanations for this difference: an increase in apoptosis only in the SGZ, an accelerated maturation process, an effect over an specific type of DCX cells or an affectation specifically in the marker analyzed, DCX, in this model.

The analysis of the number of apoptotic cells, using the marker cleaved-caspase 3, revealed a reduction in the number of apoptotic cells in both the olfactory bulb and the dentate gyrus of the hippocampus. Previous studies have shown controversial results. The analysis of P6 animals revealed no changes in the number of apoptotic cells (Lorenzi and Reeves, 2006). However, 12 month old Ts65Dn display lower number of apoptotic cells in the SVZ (Bianchi et al., 2010b). Our results are in accordance with the data observed in old animals, however the reduction is similar in the SGZ and the OB. An alternative explanation could be an accelerated maturation process or affectation of an specific subtype of DCX positive cells (Plümpe et al., 2006). This explanation must be discarded because of the results observed with other markers for immature neurons, such as PSA-NCAM or NeuroD. Using these markers, there is only a reduction of around 45% in the number of immature neurons, similar to the 41% observed in proliferating cells. Differences in the total number of cells between these markers are due to methodology (different sensibility of antibodies). Therefore, the loss of DCX expressing cells could be due to an alteration in the expression of this protein in the hippocampus of trisomic mice. Supporting this possibility, Ts65Dn mice (and individuals with DS) have an extra copy of the gene Dyrk1A (Gardiner et al., 2003). Dyrk1A phosphorylates tau (Liu et al., 2008) and this phosphorylation induces destabilization of the microtubules. DCX binds microtubules and their alteration can also affect its expression. This can explain the lower intensity of staining observed in the Ts65Dn DCX positive dendrites.

A surprising result from our study is that the number of surviving mature neurons after 21 days is similar in both Ts65Dn and control mice. However, our study agrees with the literature in that the number of proliferating and immature neurons are lower in trisomic mice. Therefore, the proportion of cells surviving is higher in trisomic mice than in euploid mice. Moreover, the analysis of the phenotype of the surviving cells reflects that the vast majority are neurons in the two analyzed regions (dentate gyrus and olfactory bulb) and in a similar proportion in control and trisomic. It has been pointed that NeuN is not a reliable marker for mature neurons in the glomerular layer of the olfactory bulb (Bagley et al., 2007). In our study we can overcome this inconvenient due to the fact that only around one per cent of BrdU positive cells in the olfactory bulb were negative for both NeuN and GFAP. Perhaps these cells could be neurons unmarked by NeuN but the results are similar.

There are two critical periods in adult neurogenesis when the number of dying cells is high, the first point is during the transient amplifier progenitor and neuroblast stages (1–4 days after mitosis) (Platel et al., 2010; Sierra et al., 2010), whereas the second is during the immature neuron stage (around the second week postmitosis) (Tashiro et al., 2006; Mouret et al., 2008). Thus, 21 days after mitosis the number of surviving cells must approximate the number of cells that will finally survive and integrate into the circuitry. Similar results have been observed analyzing the integration of newly born neurons in the olfactory bulb (Winner et al., 2002) Our results suggest that the total number of surviving cell remains unaltered independently of the number of proliferative cells. One possibility could be that the availability of trophic factors may be similar in both groups and as a consequence of that the number of cells able to survive would be similar in both cases. This possibility could be supported by the fact that the number of apoptotic cells is reduced in Ts65Dn mice as we mentioned above. Another possible scenario is that the lower cell density in the trisomic animals may offer higher opportunities for the newly generated cells of establish the proper connectivity and integrate.

Regarding the dentate gyrus, previous studies (Contestabile et al., 2007) have analyzed the survival rate of the cells generated in the SGZ, but in younger animals. 5′BrdU was injected at P2 and sacrifice was done at P30. In this case, there was a reduction in the number of cells surviving in the dentate gyrus and the phenotypical characterization of them reflects a higher amount of astrocytes than neurons. It must be taken into account that the vast majority of granule neurons of the dentate gyrus are produced during the first two postnatal weeks (for a review see Seress, 2007). Thus, the results observed in that study cannot be compared with our study at 4–5 months.

In the case of the olfactory bulb, previous studies (Bianchi et al., 2014) have observed a reduction in the survival rate, but only in old animals (13 months old). Therefore, the reduction in cell survival in the olfactory bulb observed in that study correlates with impairment in the olfactory function in these mice (de Souza et al., 2011), but can be the effect of premature aging. Similar impairment in odor discrimination has been observed in elderly individuals with DS (Nijjar and Murphy, 2002). On the contrary, in our animals (4–5 month old) cell survival is similar to controls.

### Possible consequences of the impairment in adult neurogenesis

The cognitive impairment observed in Ts65Dn mice suggest that the hippocampal function is impaired. A possible explanation for this alteration can be impairment in neuronal structural plasticity (neurogenesis, synaptogenesis and neuritogenesis). Thus, the reduction in neurogenesis observed in this study shows that these processes are affected in adult trisomic mice. However, during adulthood the number of new neurons incorporated is similar to controls. Therefore, is probable that other aspects of structural plasticity are involved more directly in the impairment observed in cognitive performance. Some studies have treated to increase cell proliferation in Ts65Dn during the early period of life. The use of fluoxetine (Clark et al., 2006; Bianchi et al., 2010a; Stagni et al., 2013) is an example of this approach. These studies were able to increase the density of proliferating cells in the SGZ and, in many cases, to improve the cognitive capabilities of Ts65Dn mice. However, it should be considered that fluoxetine increases not only cell proliferation but also the other aspects of structural plasticity such as synaptogenesis and neuritogenesis (Varea et al., 2007a,b; Guirado et al., 2012) and also increases the activity of many neurons in the brain (Guirado et al., 2009). Therefore, we cannot exclude that the improvement in cognitive performance observed as a consequence of fluoxetine treatment could be due to changes in cellular activity and synaptic and dendritic remodeling rather than to the increase in cell proliferation.

Cell proliferation in the hippocampus has been shown to be dependent of extracellular glutamate (Nácher et al., 2007). Since in DS models there is an excess of inhibition, the lower cell proliferation may be due to lower levels of extracellular glutamate in the neurogenic niches. If this hypothesis is correct, adult neurogenesis would be a consequence of the level of activity of the system (Piatti et al., 2011). DS individuals and the Ts65Dn mice model showed overinhibition in several regions of the brain such the somatosensory cortex (Pérez-Cremades et al., 2010) and the hippocampus (Belichenko et al., 2004; Hernández-González et al., 2015), Thus, overinhibition would lead to a decrease in the level of activity of the system and consequently to a reduction in cell proliferation (as we have observed in this study).

Although there is a reduction in the number of proliferating cells in trisomic mice, the final number of neurons integrated in the system is the same. Therefore, the reduction of adult proliferation cannot be held responsible for neuronal hypocellularity in the hippocampus or for the olfactory learning deficit.

## Author contributions

RL performed the experiments, discussed results and participated in the writing of the manuscript. RB performed the experiments, discussed results and participated in the writing of the manuscript. JV performed the experiments, discussed results and participated in the writing of the manuscript. JB discussed results and participated in the writing of the manuscript. CC discussed results and participated in the writing of the manuscript. JG performed the genotyping of mice, discussed results and participated in the writing of the manuscript. JN discussed results and participated in the writing of the manuscript. EV designed the experiments, discussed results and participated in the writing of the manuscript.

### Conflict of interest statement

The authors declare that the research was conducted in the absence of any commercial or financial relationships that could be construed as a potential conflict of interest.
